# Chemical shift assignments of the catalytic domain of *Staphylococcus aureus* LytM

**DOI:** 10.1007/s12104-023-10161-3

**Published:** 2023-11-02

**Authors:** Helena Tossavainen, Ilona Pitkänen, Lina Antenucci, Chandan Thapa, Perttu Permi

**Affiliations:** 1 Department of Biological and Environmental Science, University of Jyvaskyla, Jyvaskyla, Finland; 2 Department of Chemistry, Nanoscience Center, University of Jyvaskyla, Jyvaskyla, Finland; 3 Institute of Biotechnology, Helsinki Institute of Life Science, University of Helsinki, Helsinki, Finland

**Keywords:** Antimicrobial resistance, LytM, Peptidoglycan hydrolase, *Staphylococcus aureus*

## Abstract

*S. aureus* resistance to antibiotics has increased rapidly. MRSA strains can simultaneously be resistant to many different classes of antibiotics, including the so-called “last-resort” drugs. Resistance complicates treatment, increases mortality and substantially increases the cost of treatment. The need for new drugs against (multi)resistant *S. aureus* is high. M23B family peptidoglycan hydrolases, enzymes that can kill *S. aureus* by cleaving glycine-glycine peptide bonds in *S. aureus* cell wall are attractive targets for drug development because of their binding specificity and lytic activity. M23B enzymes lysostaphin, LytU and LytM have closely similar catalytic domain structures. They however differ in their lytic activities, which can arise from non-conserved residues in the catalytic groove and surrounding loops or differences in dynamics. We report here the near complete ^1^H/^13^C/^15^N resonance assignment of the catalytic domain of LytM, residues 185–316. The chemical shift data allow comparative structural and functional studies between the enzymes and is essential for understanding how these hydrolases degrade the cell wall.

## Biological context

*Staphylococcus aureus* is a pathogen of great concern because of its ability to cause life-threatening infections and its increasing resistance to antibiotics. Methicillin-resistant *S. aureus*, MRSA, causes infections hard to treat, but strikingly, MRSA strains with concomitant resistance to many other commonly used groups of antibiotics have emerged. Most alarmingly, MRSA resistance to vancomycin, linezolid, ceftaroline and daptomycin, the last-resort drugs approved for the treatment of MRSA, has been reported (Hiramatsu 1998; Tsiodras et al. 2001; Mangili et al. 2005; Nigo et al. 2017). To treat (multi)resistant bacterial infections new cures are urgently needed.

Lysins represent a novel group of potential antibacterial agents with a new mechanism of action. Lysins are naturally occurring bacterial cell wall hydrolyzing enzymes (peptidoglycan hydrolases, PGHs), which when engaged in therapeutics induce bacteriolysis (Schuch et al. 2022). PGHs are classified according to the specific type of bond they cleave. PG endopeptidases hydrolyze bonds within the peptidic moieties in the bacterial PG, which in *S. aureus* consist of two stem peptides (Ala-D-iso-Gln-Lys-D-Ala) crosslinked by pentaglycine cross-bridges. The latter is the target of the glycyl-glycine endopeptidase LytM, one of *S. aureus* autolysins (Ramadurai et al. 1999).

We have recently assigned the chemical shifts of the LytM N-terminal domain and the linker region, encompassing residues 26–184, for the characterization of its structure and interactions (Pitkänen et al. 2023). LytM catalytic domain (LytM CAT, residues 185–316), is structurally homologous to lysostaphin and other MEROPS M23B family of metallo-endopeptidase catalytic domains (Firczuk et al. 2005; Grabowska et al. 2015). These enzymes have in common a characteristic narrow groove formed by a β-sheet and four surrounding loops. At one end of the groove, a catalytic zinc ion is coordinated by two conserved histidines and an aspartate. The Zn^2+^ ion, which polarizes the peptide bond, and a nucleophilic water molecule activated by two other conserved histidines act in concert to hydrolyze the substrate glycyl-glycine bond (Grabowska et al. 2015).

Lysostaphin catalytic domain is more active than LytM CAT in *S. aureus* bacterial lysis (Osipovitch and Griswold 2015). LytM CAT in turn defeats LytU, another *S. aureus* M23B autolysin (Raulinaitis et al. 2017a, b), in exogenous bacteriolytic activity (Antenucci et al. unpublished data). Also, in vitro, the preferred Gly-Gly target bond seems to differ between the three enzymes, although comparison is not straightforward because of the nature of substrates, sample conditions and techniques (Xu et al. 1997; Odintsov et al. 2004; Warfield et al. 2006; Raulinaitis et al. 2017b). Our recent study, in which we used identical conditions and techniques for lysostaphin and LytM, revealed similarities but also differences in their target bond specificity and substrate hydrolysis rates (Antenucci et al. 2023). Indeed, our goal is to compare and understand how differences in structure and dynamics can give rise to functional dissimilarities, which is essential in the development of PGHs into potent antimicrobials. To this end, LytM CAT chemical shift assignments, together with those of lysostaphin and LytU (Raulinaitis et al. 2017a; Tossavainen et al. 2018) allow comparative structural, dynamical and interaction studies.

## Methods and experiments

### Expression and purification of LytM CAT

The *S. aureus* LytM catalytic domain (residues 185–316) was cloned into pGEX-2T plasmid and overexpressed in *Escherichia coli* strain BL21(DE3) pLysS as a glutathione S-transferase (GST)-fusion protein with a thrombin cleavage site. To produce uniformly ^15^N and ^13^C labelled protein, the cells were grown in standard M9 minimal medium supplemented with 100 µg/ml ampicillin, ^15^NH_4_Cl (1 g/l) and ^13^C-D-glucose (2 g/l) as the sole nitrogen and carbon sources, respectively. Briefly, overnight bacterial preculture was expanded to two liters and cells were grown at 37 °C, 250 rpm until the OD at 600 nm reached 0.6. Then protein expression was induced by adding 0.5 mM isopropyl β-D-1-thiogalactopyranoside (IPTG) and cells were incubated at 25 °C, 250 rpm for 16 h. Cells were harvested by centrifugation, resuspended in phosphate-buffered saline (PBS) buffer and lysed using EmulsiFlex-C3 high-pressure homogeniser (Avestin). Protein was captured using Protino Glutathione Agarose 4B (Macherey-Nagel) according to manufacturer’s instructions. GST was cleaved in situ using thrombin protease (BioPharm Laboratories, LLC). Cleaved protein was eluted and further purified by size exclusion chromatography using ÄKTA pure chromatography system (GE Healthcare) with HiLoad Superdex S75 (16/60) column (GE Healthcare) in 20 mM sodium phosphate pH 6.5, 50 mM NaCl buffer. Protein was concentrated using Amicon Ultra-15 centrifugal filter units (Millipore).

### NMR spectroscopy

0.4 mM LytM catalytic protein preparation, uniformly ^15^N, ^13^C labelled in 20 mM sodium phosphate (pH 6.5), with 50 mM NaCl, 0.6 mM ZnCl_2_ and 95% H_2_O/5% D_2_O was used for resonance assignments. Protein backbone resonances were assigned by analyzing HNCACB, HN(CO)CACB (Yamazaki et al. 1994), HNCO (Muhandiram and Kay 1994), i(HCA)CO(CA)NH (Mäntylahti et al. 2009), HBHA(CO)NH spectra, whereas aliphatic and aromatic side chain assignments were obtained from H(CCO)NH, (H)C(CO)NH, HCCH-COSY, and HB(CBCGCD)HD, HB(CBCGCDCE)HE, ^1^H-^15^N and ^1^H-^13^C NOESY spectra (reviewed in Sattler et al. 1999), respectively. Assignment of methyl-containing residues was accomplished with the DE-HCCmHm-TOCSY experiment (Permi et al. 2004).

The sample was subsequently exchanged into 100% D_2_O, and the order of disappearance of amide peaks was followed by measuring ^1^H-^15^N HSQC spectra. From this sample another set of aliphatic and aromatic region ^1^H-^13^C NOESY spectra, as well as 4D HACACON (Tossavainen et al. 2020) and 4D HACANCOi (Karjalainen et al. 2020) spectra were acquired.

All NMR experiments were performed at 298 K on a Bruker Avance III HD 800 MHz spectrometer equipped with a ^1^H, ^13^C, ^15^N cryogenic TCI probe. NMR data were processed using Topspin (Bruker) and analyzed using CcpNmr Analysis v. 2.5.2 (Vranken et al. 2005).

## Extent of assignments and data deposition

**Fig. 1:**
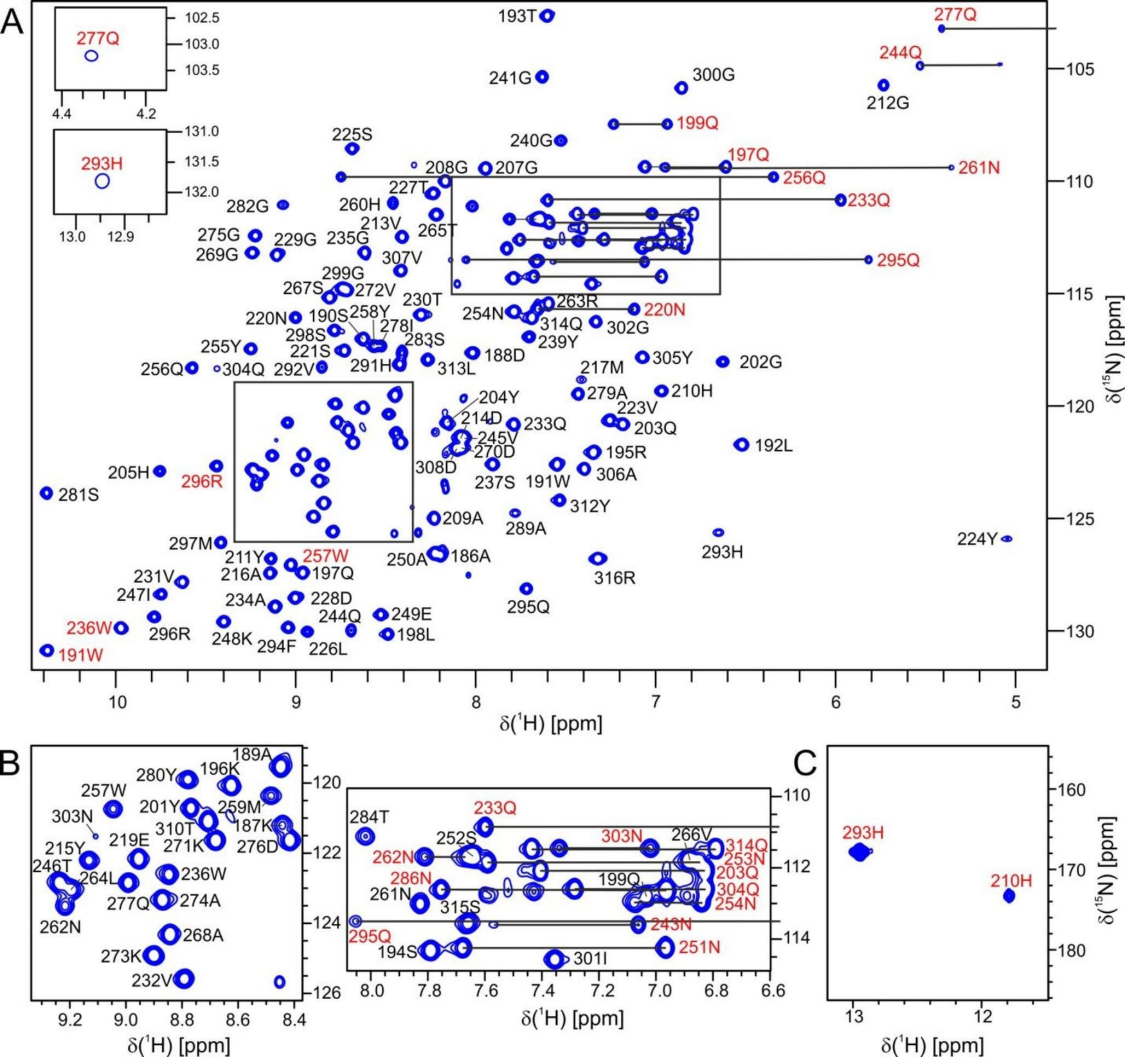
NMR resonance assignments of LytM CAT. **a**^1^H-^15^N HSQC spectrum of LytM CAT recorded at 800 MHz ^1^H frequency, 298 K. The upper inset shows the peak of Gln277 side chain Hε21, which has an unusual upfield chemical shift, 4.33 ppm. The lower inset shows the ε2 side chain peak of H293, one of the zinc-coordinating residues. The peak is folded, and its true ^15^N chemical shift is 167.8 ppm, see panel c. The low-intensity peaks in the middle ^1^H region of the spectrum arise from a small amount of unfolded protein present in the sample. Red labels indicate side chain peaks. **b** Enlargements of the crowded regions indicated with boxes in panel a. **c** The δ1 side chain peak of the other zinc-coordinating histidine is visible in a ^1^H-^15^N HSQC spectrum in which the ^15^N transmitter frequency was set to histidine side chain protonated nitrogen region, 170 ppm. The intensity of H210 peak is five times lower than that of H293, which explains why it is not present in the traditional ^1^H-^15^N HSQC spectrum.

LytM CAT ^1^H-^15^N HSQC spectrum displays very well dispersed peaks with a few peaks with noteworthy upfield chemical shifts. Y224 amide proton and side chain ε2 protons of Q244, Q277 have shifts below 5.2 ppm, which is consistent with these interacting with aromatic side chains as seen in the crystal structure of LytM CAT (Firczuk et al. 2005). The good dispersion of peaks in LytM CAT spectra in general arises from the almost all-beta fold and the large number of aromatic residues in the amino acid sequence (1 Phe, 6 His, 3 Trp, and an enriched amount of tyrosines, 11). Of note are the side chain N-H peaks of the two zinc-coordinating histidines, H210 and H293 (Fig. 1c). These are likely to be visible because metal coordination locks their tautomeric state, and additionally both are hydrogen-bonded to nearby residues, H210 δ1-P200 O and H293 ε2-Q295 Oε1, in the crystal structure (Firczuk et al. 2005). The corresponding peaks were visible also in the HSQC spectra of lysostaphin and LytU.

However, peak intensities show significant variation, and sixteen amide peaks have broadened beyond detection. In addition to the N-terminal residues 183–185, likely to be unstructured in solution, G206, N238, G242-N243, N251, N253, G285-T288, S311 do not show a backbone peak in the ^1^H-^15^N HSQC spectrum. Seven of these eleven amides are located in the loops surrounding the catalytic groove. Notably four consecutive residues in the ten-residue loop between strands β7 and β8, which borders the catalytic histidines H260 and H291 are not observed. Apart from the N-terminal residues, most of the unassigned side chain resonances are found within this same loop and the catalytic histidines. The assignment percentages are the following: ^1^H^N^ 88% (115 out of 132 non-proline residues), ^15^N 91% (125 out of all 137 residues), ^13^Cα 96% (131/137), ^13^Cβ 97% (114 out of 118 non-glycine residues), and ^13^CO 93% (127/137) for backbone resonances and 98% for aliphatic and 90% for aromatic side chain resonances. The ^1^H, ^15^N, ^13^C chemical shift assignments for LytM CAT have been deposited in the BioMagResBank (http://www.bmrb.wisc.edu) under accession number 52149.

**Fig. 2:**
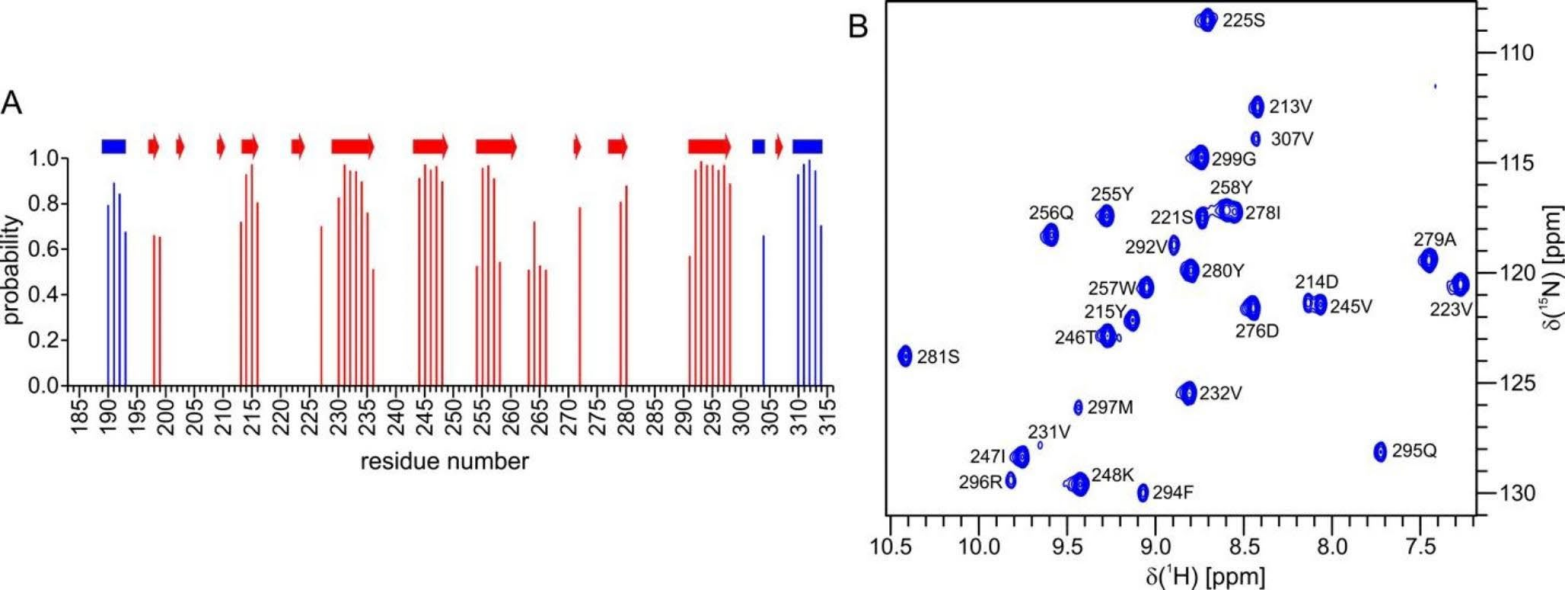
Secondary structure prediction and amide protons protected from exchange. **a** Secondary structure prediction by TALOS-N, with blue bars representing helices and red bars strands. On the top is depicted the secondary structure present in LytM CAT crystal structure (PDB ID 2B13, chain A), with blue rectangles indicating helices and red arrows strands. **b**^1^H-^15^N HSQC displaying amide peaks protected from exchange. The spectrum was acquired ~ eight days after lyophilized LytM CAT had been dissolved in D_2_O.

Although signal dispersion convincingly suggests a well-folded and stable protein in the current sample conditions, we further studied its properties by determining its secondary structure based on assigned chemical shifts using TALOS-N (Shen and Bax 2015), and by evaluating hydrogen-to-deuterium (H/D) exchange rates. The secondary structure predicted by chemical shifts well reproduces that observed in the crystal structure, except for the missing short β strands (G202-Q203, A209-H210, P222-Y224, A306-V307) and the predicted strand for residues R263-V266 (Fig. 2a). In the crystal structure R263 and T265 show strand-like hydrogen bonding, but T265 ψ angle does not conform to that in a canonical β strand.

The H/D exchange spectra indicate that LytM CAT has a well-protected core, which resists exchange. After approximately eight days in D_2_O, 28 amide LytM CAT peaks are still present in the ^1^H-^15^N HSQC spectrum (Fig. 2b). Except for R296 N-H all these amides are hydrogen bonded, 22 of them in strands and five in residues flanking strands. The persistence of R296 N-H is likely to be explained by its hydrogen bond to an intramolecular H_2_O molecule, which in total is stabilized by four hydrogen bonds. In all, LytM CAT in solution appears to faithfully replicate the structure determined by X-ray crystallography (Firczuk et al. 2005).

## Data Availability

The chemical shift assignments have been deposited to the BMRB under the accession code: 52,149.
